# Implication of the Strand-Specific Imprinting and Segregation Model: Integrating *in utero* Hormone Exposure, Stem Cell and Lateral Asymmetry Hypotheses in Breast Cancer Aetiology

**DOI:** 10.4172/2161-1041.s2-005

**Published:** 2013-08-13

**Authors:** Singh Harbinder, Carol A Lazzara, Amar JS Klar

**Affiliations:** 1 Department of Biological Sciences, Delaware State University, Dover, USA; 2 Gene Regulation and Chromosome Biology Laboratory, Frederick National Laboratory for Cancer Research, National Institutes of Health, Frederick, USA

**Keywords:** Breast cancer aetiology, *In-Utero* hormone exposure, Immortal strand hypothesis, Asymmetric chromatid segregation, Cerebral laterality

## Abstract

Known genetic mutations and familial hereditary factors account for less than 20–25% of breast cancer cases in women, therefore, most instances have been classified as sporadic cases of unknown aetiologies. Single nucleotide polymorphisms (SNPs) were considered as breast cancer risk factors, but numerous studies have failed to support this assertion. Recent evidence correlates aberrant epigenetic mechanisms in the development and metastatic progression of breast cancer, yet there has been limited progress made to identify the primary aetiology underlying sporadic cases of breast cancer. This has led some researchers to consider alternative hypotheses including *in utero* exposure to deleterious chemical agents during early development, the immortal strand and the strand-specific imprinting and selective chromatid segregation hypotheses. Here, we integrate prominent alternate models to help guide future research on this very important topic concerning human health.

The aetiology of cancer, including breast cancer, is often discussed in the context of genetic and environmental risk factors. Skin cancer is the major class of cancer that occurs both in men and women and its risk factor is predominantly considered to be environmental exposure to sun. On the other hand, genetic risk factors are considered key to the development of breast cancer, the second most common cancer, accounting for nearly one in three cases diagnosed in United States women [1]. The American Cancer Society lists some of the perils in breast cancer as non-modifiable, such as gender (women account for ~99% of the cases), race (Caucasian women have the highest risk), early menses (increased risk if menses begin <12 years age), and increased risk with age. Others are modifiable factors—use of oral contraceptives, hormone replacement therapy, obesity, and use of alcohol—all of which contribute to increased risk of breast cancer. Even though BRCA1 and BRCA2 account for the majority of genetic mutations found in breast cancer cases, only 5–10% of breast cancer cases result directly from specific gene mutations. Another 15–20% of cases are considered to be familial, such that a cluster of cancers affects first- or second-degree relatives, but with an unclear inheritance pattern [2]. The majority of breast cancer cases are termed “sporadic” cancers, with apparently no known cause; however researchers have suggested numerous possibilities over the past several decades. Here we provide an overview of the progression of ideas proposed for explaining the cause of breast cancer, followed by our perspective aimed at suggesting an aetiology that integrates some of the more plausible concepts.

According to the American Society of Clinical Oncology, autosomal dominant mutations of the *BRCA1* and *BRCA2* genes with low penetrance act as predisposing factors for hereditary breast/ovarian cancer (HBOC); that is, not all subjects carrying disease susceptibility mutations even in homozygous conditions develop disease. These mutations are also associated with an increased incidence of pancreatic, colon, and prostate cancers in men [3]. Individuals with inherited mutations in several other genes, e.g., *TP53, PTEN, STK11,* and *CDH1,* may also have an increased risk of developing breast cancer, but these genetic defects account for a very small percentage of cases[4,5]. For example, a germ line mutation in TP53 is associated with 50–60% increased risk of Li-Fraumeni syndrome, a type of breast cancer by age 45, yet the syndrome itself is very rare (1 to 9 in 10,000) [6]. Characteristics of familial cancers include prevalence of disease in two or more affected first or second-degree relatives, later onset of disease, unilateral occurrence, but by an unclear inheritance pattern not yet understood by applying the rules of simple genetics [4]. Overall, familial cancer clusters provide an approximately two-fold increase in breast cancer risk over that of the general population. The contributory familial cancer genes *CHEK2, ATM, NBS1, RAD50, BRIP1,* and *PALB2* have been shortlisted based on subtle sequence variants or polymorphisms that could be associated with a small to moderate increased risk for breast cancer [4].

Single Nucleotide Polymorphisms (SNPs) make up about 90% of all human genetic variation, with numerous SNPs linked to various diseases including breast cancer. For example, three SNPs of the MBD2 transcriptional repressor gene are associated with increased breast cancer risk yet another three seem to offer marginal protection [7]. The role of SNPs in the cancer process has been extensively investigated, though little agreement has been reached to implicate specific SNPs in the disease aetiology. Several consortia e.g., the California Teachers Study Cohort [8] and the National Cancer Institute Breast and Prostate Cancer Cohort Consortium [9] have focused on establishing links between genetic variants and breast cancer susceptibility. A large multistage study for susceptibility alleles identified four novel suspect genes, yet the results also revealed that a high proportion of the general population carries susceptibility SNPs without developing the disease and that the increased risk associated with these alleles is relatively small [10]. However, studies based on breast cancer susceptibility SNPs from genes involved in major cancer-related pathways concluded that there was statistically significant evidence for gene-gene (SNP-SNP) interaction with concomitant increased breast cancer risk [11,12]. In contradiction, results from another elaborate study did not support the view of interaction between suggested breast cancer susceptibility loci and established risk factors [13].

Having failed to discern the aetiology of sporadic cases by familial genetics and genome-wide SNP mapping studies, researchers have searched for other causes. There has been relatively recent recognition that epigenetic factors may be significant in the development and progression of cancers. DNA methylation of CpG islands in promoter regions can generally remodel chromatin. Both hypermethylated and hypomethylated regions in the genome have been proposed to exert epigenetic influences in breast cancer [14]. Promoter methylation caused by overactive DNA methyltransferases has been implicated in the silencing of ~75 key tumor suppressor genes (TSG) related to breast tumor genesis; these include genes for cell cycle regulation, DNA repair, breast cancer (*BRCA1, BRCA2*), cell-signaling pathways, and estrogen-α and progesterone receptors [15]. Abnormal histone modification, in combination with DNA hypermethylation, has been associated with epigenetic silencing of TSGs and genomic instability in breast cancer [16]. Histone deacetylase (HDAC) inhibitors are known to induce cell death, as well as impede proliferation of cancer cells, by regulating cell cycle genes by as-yet-unknown mechanisms [17]. In addition, micro-RNAs (miRNAs) have been reported to exert their influence in breast cancer by regulating either tumor suppressor genes or oncogenes [18]. Diminished miRNA expression associated with CpGhypermethylation was observed in breast cancer tissue [19]. In a study of 76 breast cancer and 10 noncancerous control specimens, researchers were able to distinguish the difference between cancerous and control specimens 100% of the time based on observations of the down- or up-regulation of very specific miRNAs [20]. We surmise that such studies have only tabulated molecular events associated with the progression of the disease rather than discovering the initial cause of it. Comprehensive and current reviews on the genetics and epigenetics of cancer are available elsewhere [21–23].

The immense amount of information unearthed in the study of cancer in general, and breast cancer in particular, indicates that allelic and epigenetic factors are intertwined in complex mechanistic interactions. Advancements in the field, including technological innovations, are expected to resolve these interactions towards a meaningful understanding of breast cancer. It is justifiably contended that there has been limited progress in understanding the basis of the disease [24]. Notably, the aetiologies of sporadic cases that account for the overwhelming majority of breast cancer cases remain unknown. Some researchers have advocated alternative developmental mechanisms to explain the causes of sporadic cases. Two of these models propose *in utero* changes and an increased stem cell population as the keys to understanding breast cancer [25–27]. A third model that considers the chromosomal basis of anatomic laterality development may help bridge the gap between the previous proposals [28–29].

## In-utero Hormonal Exposure and Subsequent Breast Cancer Development

There have been many, often conflicting, studies about estrogen vis-à-vis breast cancer tumorigenesis and metastases. In 2003, the National Institute of Environmental Health Sciences (NIEHS) added estrogen to its list of known cancer-causing agents [30]. However, according to a recent 2012 report based on a large Women’s Health Initiative study, postmenopausal women who took estrogen alone had a lower incidence of invasive breast cancer than those who received a placebo [31]. Diethylstilbestrol (DES) is a synthetic form of estrogen that was prescribed for pregnant women between 1940 and 1971 to prevent miscarriage, premature labor, and related complications of pregnancy. DES is considered an endocrine-disrupting chemical (EDC), which alters endocrine function due to its hormone-like activity. According to the American Cancer Society, women who took DES during pregnancy increased their risk of breast cancer by 30% over women who were not exposed to the drug. Daughters exposed to DES *in-utero* are at increased risk of developing clear-cell adenocarcinoma (CCA) of the vagina and cervix as well as structural abnormalitiesin the anatomy of the reproductive tract [32]. In addition, they can experience vaginal epithelial changes and may have fertility problems and pregnancy complications. Sons exposed to the drug *in utero* have been shown to experience epididymal cysts, microphallism, and cryptorchidism, among other abnormalities [33]. However, there has not been any clear evidence that DES exposure has contributed to an increased cancer risk in men [34]. In addition to the increased risk of vaginal and cervical CCA, there is evidence that DES exposure *in utero* may be linked to breast cancer susceptibility. A large-cohort epidemiological study showed that women with prenatal DES exposure have an increased risk of breast cancer after 40 years of age [35]. In 2011, a follow-up study of 4,653 women exposed *in utero* DES and an unexposed control group of 1,927 women was undertaken to assess the risks of 12 adverse outcomes linked to DES exposure. The cumulative risk for breast cancer in DES-exposed and -unexposed women was 3.9% and 2.2%, respectively [36].

Bisphenol-A (BPA), a chemical used in many commonly used plastics, is another known EDC. BPA and DES are nonsteroidal, display similar chemical structures, and have estrogenic effects. Both DES and BPA have been shown to interfere with the developmental programming of breast tissue in mice (*inutero*), as well as in the human breast adenocarcinoma cell line MCF-7 [37]. Exposure to DES and BPA caused increased expression of a specific methyltransferase in the mammary gland that has been associated with breast cancer invasion and progression [38]. Another study showed that prenatal exposure of rats to BPA results in neoplasias (carcinoma *in situ)* in the mammary glands of 33% of the exposed rats compared to none in unexposed fetuses. In addition, animals developed mammary tumors when a sub carcinogenic dose of a chemical carcinogen was administered to rats at puberty [39]. These observations provide specific pieces of evidence, although indirect, that link the *in utero* hormonal exposure with an increased risk of breast cancer of the progeny. We suggest that such experimental manipulations possibly interfere with normal biological mechanisms to cause increased disease incidence, and therefore these studies should not necessarily be construed to favor *in utero* hormonal exposure being the cause of sporadic cases found in women at large.

## Stem Cells and the Immortal Strand Hypothesis

Another idea considers possible fetal growth influences on breast cancer development and proposes that the number of mammary-specific stem cells, which is determined *in utero* or soon after birth, correlates with the likelihood of developing breast cancer [25–26]. The stem cell hypothesis holds that cancers originate in stem or progenitor cells with stem cell properties [40]. The risk for cancer development may be directly related to the size of the stem cell pool and its mitotic activity. Radiologic studies have shown a significant association between breast density and the risk of breast cancer including stromal fibrosis of the breast [41,42]. It has been proposed that breast mass correlates with the total number of mammary cells and, as a corollary, to the number of mammary stem cells [26]. This has been associated with higher levels of estrogen and IGF-1 exposure *inutero* or in the perinatal period [26,27]. However, it has also been pointed out that women with normal breast density also develop breast cancer and women with higher breast densities may not show disease symptoms [42]. Furthermore, high heritability in breast density has been observed in monozygotic twins [43]. In a wide-ranging model, it has been suggested that the correlation between breast cancer and breast density does not represent a causative relationship but is rather due to genetic linkage [44,45].

In 1975, John Cairns put forth a hypothesis to elucidate possible function of previously reported observations of non-random sister chromatid segregation [46,47]. His immortal strand hypothesisposits that stem cells divide by asymmetric cell division, with one daughter cell ordained to become stem cell while the other fated to differentiate, by an unknown mechanism. According to this theory, all chromatids containing the oldest template strands are delivered by the parent cell to the stem-cell daughter, while all chromatids containing the first-time employed template strands are delivered to the differentiating daughter cell. In theory, this will allow stem cells to escape from inheriting potential cancer-causing DNA replication errors and thereby retain the immortal strands throughout an organism’s life [46–48]. This hypothesis has continued to evoke the interest of researchers but has been difficult to prove or invalidate definitively. A recent report on asymmetric histone distribution on segregating sister chromatids in cultured mammalian cells offers indirect support to the hypothesis and the assay used in this study holds potential for use in other systems, including embryonic tissue [49]. Another study has shown that labeled template strands are retained in one daughter cell during division termed as the label-retaining epithelial cell (LREC), while newly synthesized DNA strands are preferentially distributed to the other daughter cell in mouse mammary glands [50]. A similar labeling study in mice, implanted with premalignant progenitor cells derived from murine mammary epithelial cells showed that sub-populations of LRECs expressing estrogen-α and progesterone receptors retained their original premalignant DNA strands, while the LRECs preferentially distributed the second label to daughter cells [51–53]. Additional studies have also shown that stem cells from skeletal muscle [54], cardiac progenitor cells [55–56]as well as other diverse systems [57–60] exhibit biased chromatid distribution. On the contrary, others have observed biased segregation of only copies of X and Y chromosomes during stem cell division in *Drosophila* [61]. Critics of the immortal strand hypothesis argue that even if it were presumed that immortal strands exist, they would still be vulnerable to mutations and recombination, thereby losing their postulated ability to form new stem cells [62]. Furthermore, it has been technically difficult to establish that biased segregation indeed concerns all chromosomes, as originally envisioned by Cairns, rather than a subset of chromosomes.

A significant implication of the immortal strand hypothesis is that sister chromatids are genetically different from one another and are imprinted to undergo asymmetric, strand-specific segregation during mitosis. In principle, biased distribution of only a few select chromosomes would be sufficient to accomplish cell-type specific functions, such as cellular differentiation or laterality development, and will not necessarily be limited to maintaining stem cell populations. This point remains important in the context of the information discussed in the next section, where selective segregation of epigenetically distinct sister chromatids is proposed as a molecular mechanism for generating the asymmetric cell division, an essential requirement for developmental precision.

## Cerebral Laterality and Breast Cancer

The cerebral hemispheres of the brain are anatomically and functionally asymmetrical, similar to visceral organ asymmetry in humans [63–65]. Imaging studies on live subjects have reported reversed or atypical cerebral asymmetry in patients with neurological disorders such as developmental dyslexia [66] and autism [67] as well as in a non-neural disorder, namely breast cancer [68]. Reports associating atypical cerebral asymmetry with breast cancer contend that both biological conditions are functions of abnormal hormonal exposure *in utero* [25, 69,70]. To test this idea, researchers analyzed the correlation between breast cancer occurrence and brain laterality using cranial computed tomography (CT) scans of right-handed women with breast cancer and of healthy right-handed subjects as controls [68]. Typical asymmetries seen for right-handed women include: the right frontal area measuring wider and the right frontal pole protruding more anteriorly than the left, and the left occipital pole both measuring wider and protruding more posterior than the right [63]. Remarkably, the results revealed that women with breast cancer had a higher incidence of reversed cerebral asymmetry: a 49% reversal of brain laterality compared to 18% found in the unaffected individuals. Since brain asymmetry was thought to be a likely consequence of abnormal *in utero* hormonal exposure, the results were interpreted to support the *in utero* hormone exposure breast cancer model put forth previously [25]. In a postscript within their original report, Sandson and co-workershad noted that unidentified genetic factors in the embryo might provide alternative explanations for their observations [68].

**Figure 1:**
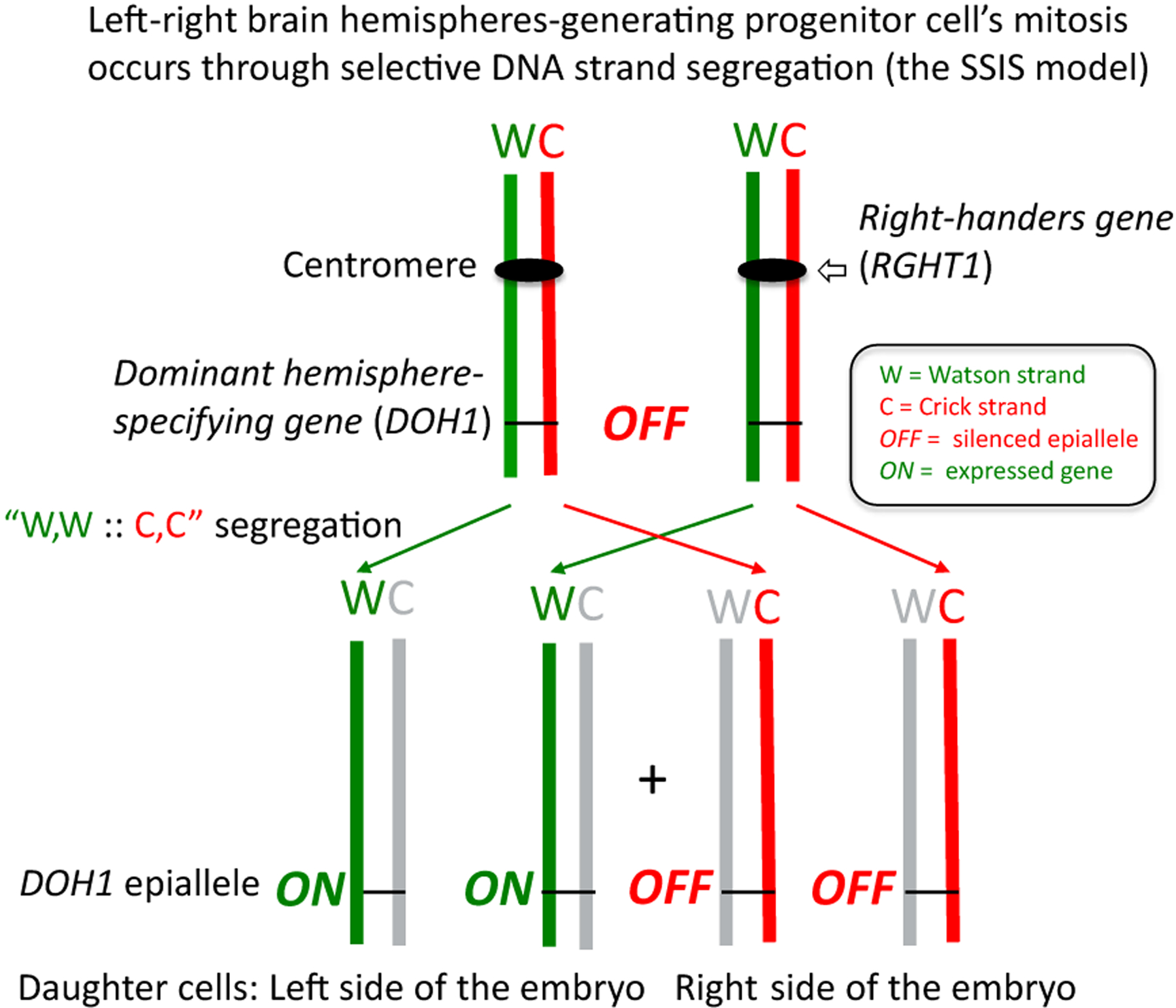
The somatic strand-specific imprinting and segregation (SSIS) model: The SSIS model was proposed to produce developmentally nonequivalent daughter cells of the cerebral hemisphere laterality generating progenitor cell in embryogenesis. One of the daughter cells, the one placed on the left side of the embryo (with respect to anterior-posterior and dorso-ventral axes), inherits specific chromatids of both homologs of Chromosome 11, those containing the template “Watson” (W) strand with the expressed (*ON*) epiallele of the hypothetical language-process specifying gene (*DOH1*) gene. The daughter cell on the right side of the embryo inherits indicated chromatids/strands with epigenetically silenced *DOH1* gene’s epialleles. A hypothetical *RGHT1* (right-handedness-specifying gene) directs biased segregation of differentiated sister chromatids by functioning at the Chr. 11 centromere. The DNA strands are color-coded to indicate their biased distribution to specific daughter cells. Grey colored strands reflect “younger” strands synthesized in the parental cell. Due to biased strands “W,W::C,C” segregation occurring in mitosis, differentiated daughter cells result through asymmetric cell division, and after subsequent growth, differentiated brain hemispheres develop such that a person processes language in the left hemisphere of the brain. In contrast, homozygous individuals containing the nonfunctional allele of the *RGHT1* gene, *r*/*r* (*r* for *r*andom), lack biased segregation and therefore exhibit random hemispheric asymmetry distribution. It is suggested that predisposition to breast cancer only in a minority of *r*/*r* individuals results from stochastic errors of the SSIS process in breast tissue stem cells, including allowing rare somatic recombination events to cause loss-of-heterozygosity of imprints of tumor suppressor genes.

After remaining largely ignored for almost two decades, data from the above study were explained by employing a different hypothesis [29]. According to this new interpretation based on a random-recessive model [28], a postulated gene (*RGHT1*) is responsible for coupling the development of right- versus left-hand-use preference for unimanual tasks (i.e., handedness) and brain hemispheric structural and functional asymmetry (Figure 1). However, in individuals with non-functional recessive allele in the homozygous condition (*r*/*r* genotype), uncoupling of the brain laterality and handedness traits occurs so that they are distributed randomly and independently from one another to the left- or right-side of an individual. By investigating only right-handed women subjects with breast cancer, Sandson and coworkersmade a truly remarkable observation in that there was 49% incidence of brain laterality reversal in breast cancer patients [57]; in other words, cancer subjects exhibited random distribution (~50:50) of brain laterality. The nervous system and the integument (including mammary glands) arise from ectodermal cells during embryonic development. The observation of nearly 50% brain laterality reversal in breast cancer patients clearly suggests that they possess the *r*/*r* genetic constitution according to the random-recessive model. Originally, the *RGHT1* gene was proposed to promote asymmetric cell division for establishing brain laterality. It was also proposed that breast cancer may be caused by random errors in asymmetric cell division in some *r*/*r* individuals. Specifically, the *r*/*r* genotype may cause Loss of Heterozygosity (LOH) through increased mitotic recombination operating later in adult life to cause breast disease [29]. We suggest that *r*/*r* genotype increases the rate of mitotic recombination, as compared to that of the *R*/*R* and *R*/*r* genotype, leading to LOH across the genome, including chromosome regions where genes mutations or TSGs implicated in breast cancer development lie. Our thesis concerns the reason why initially cancer originates, not later biochemical events associated with cancer progression. The genetics of the Strand-Specific Imprinting and Selective chromatid segregation model (SSIS) have been described previously [28]. When LOH is associated with cancer, it has been generally assumed that recombination causes homozygosis of the non-functional, mutant or epigenetically silenced allele of a TSG gene [71,72]. However, two groups have reported frequency of LOH occurring in as many as 50% of cancer cases in regions that include well-known, cancer pre-disposing genes BRCA2, p53 and RB1 [73,74]. Furthermore, it has been reported that 50% of breast tumors showed LOH in 1 or more of the 10 microsatellite markers used in the study and that the LOH did not necessarily result in the deletion of TSGs [74]. The reported observations that 50% of sporadic breast cancer cases are associated with somatic recombination, causing LOH of regions in or around cancer genes support the suggestion of the SSIS model, where by the *r*/*r* genotype is a predisposing factor for somatic recombination in breast tissue, and separately, for brain hemispheric randomization *in utero* through asymmetric cell division. These explanations provide a new basis for the amazing association of sporadic breast cancer predisposition to brain laterality development [68]. Models for utilizing DNA strand asymmetry in cellular differentiation, cancer development or as a vehicle for controlling the speed of evolution have been recently reviewed [75].

The mechanism of body laterality development continues to remain controversial and is an exceptionally active field of research [76,77]. Considering the genetic correlates of cerebral laterality development with the predicted *r*/*r* genetic constitution [28], the latter clearly constitutes the genetic predisposition factor for sporadic cases of breast cancer. It does so by allowing increased recombination in breast tissues in chromosome regions around the genes which have been implicated in familial cases of breast cancer. Future research directed towards closer scrutiny of the remarkable association between brain laterality distribution and breast cancer is likely to discern contribution of genetics, LOH and epigenetic factors in the disease. Should this association be replicated by future studies, it will be rewarding to define the molecular intricacies of how the *r*/*r* genotype initiates the process of carcinogenesis that is restricted to a minority of the population.

## Conclusions

The aetiology of sporadic breast cancer cases remains unknown. A number of large studies aimed at defining breast cancer aetiology using whole-genome sequencing of diseased and healthy breast tissues have unearthed thousands of genomic variations [78], but they were unable to define the fundamental reason why some individuals are susceptible while others remain unaffected. Considering the lack of success in elucidating the causes of cancer by current approaches despite commendable efforts, attention needs to be focused on alternative theories. The immortal strand hypothesis proposes that a pool of stem cells is created as a result of asymmetric cell divisions. The hypothesis that breast cancer may start *in utero* is supported by studies indicating that the number of mammary stem cells is increased by *in utero* exposure to estrogen and IGF-1. Moreover, higher breast density in breast cancer patients has been proposed to be a consequence of the increased number of stem cells in the breast tissue, yet this idea remains controversial with increasing evidence contrary to the previously suggested cause-effect relationship between breast density and cancer. Our hypothesis involves consideration of the biological consequences of laterality specifying genetics. Sandson and coworkers’ defining 1992 study showed that right-handed women with breast cancer have a remarkably increased prevalence of reversed functional laterality of the brain, as compared to right-handed subjects with typical brain laterality [68]. A previously proposed model postulated a hand-use preference gene, which, in its nonfunctional (double-recessive *r*/*r*) allelic form confers random brain hemispheric laterality during embryonic development [28]. The enhanced version of this model implies that the *r*/*r* genotype also constitutes a predisposing factor for breast cancer development in adult life [29]. Notably the handedness model and the immortal strand hypothesis are based on nonrandom segregation of sister chromatids; the former also explains the observed association of development of the functional asymmetry of the nervous system *in utero* with breast cancer development occurring later in adult life.

Clearly, further research is required to establish a definitive association between the two seemingly unrelated traits of brain laterality and breast cancer explored in our perspective. It is interesting to note that gliomas and melanomas have also been reported to correlate with atypical laterality [79–81]. Cairns’ immortal strand hypothesis was proposed as a mechanism to avoid inheriting potential cancer-causing DNA replication errors in stem cells, and it was based on the discovery of biased segregation of sister chromatids of all chromosomes in some biological systems [46]. The SSIS model invoked biased chromosomeand cell-type-specific segregation of epigenetically differentiated sister chromatids to accomplish cellular differentiation and vertebrate development [82]. Here, we highlight that the SSIS mechanism, which perhaps evolved primarily to accomplish development of hemispheric laterality, constitutes a predisposing factor for causing breast cancer in humans. Considering the discussion presented in this perspective and the recent retrospective by Karl Lark (one of the first to observe non-random chromatid segregation) on the general consequences of the “premature” discovery [83], further examination of the idea that the *r*/*r* genotype constitutes a primary cancer predisposition factor has the potential to help uncover the disease aetiology. To be noted, the existence of WW:: CC segregation pattern was recently spotted by Sauer and Klar, 2013 [84] in a study of *Drosophila* autosomes by Yadlapalli and Yamashita, 2013 [61]. The key message of our perspective points out the lack of success in identifying aetiology of breast cancer despite extensive amount of research conducted on the topic. We hope our perspective helps to guide future studies to define the aetiology of sporadic cases of breast cancer in women. It is crucial to first replicate the Sandson et al. [68] study published back in 1992.

## Abbreviations:

SNPs: Single Nucleotide Polymorphisms
EDC: Endocrine-Disrupting Chemical
IGF-1: Insulin-like Growth Factor
SSIS: Somatic Strand-Specific Imprinting and Segregation Model
